# A *Schistosoma japonicum* MicroRNA Exerts Antitumor Effects Through Inhibition of Both Cell Migration and Angiogenesis by Targeting PGAM1

**DOI:** 10.3389/fonc.2021.652395

**Published:** 2021-06-16

**Authors:** Chao Hu, Yuzhen Li, Danting Pan, Jing Wang, Liufang Zhu, Yu Lin, Shanli Zhu, Weiqing Pan

**Affiliations:** ^1^ Institute for Infectious Diseases and Vaccine Development, Tongji University School of Medicine, Shanghai, China; ^2^ Department of Tropical Diseases, Naval Medical University, Shanghai, China

**Keywords:** *Schistosoma japonicum*, microRNA, hepatoma cell, PGAM1, cross-species regulation

## Abstract

MicroRNA (miRNA) is an important regulator for gene expression. Recent studies showed that some heterogenous miRNAs derived from both parasite and plant can regulate expression of mammalian gene in a cross-species or even a cross-kingdom manner. Here, we identified a *Schistosoma japonicum* miRNA (designated as sja-miR-61) that is present in the hepatocyte of mice infected with the parasite. The sja-miR-61 mimics significantly inhibited the migration of both mouse and human hepatoma cells *in vitro*. In a xenograft animal model, significant reductions of the tumor volume and weight were observed in mice inoculated with hepatoma cells transfected with sja-miR-61 mimics compared to the controls. We found that the *in vivo* inhibition of tumor growth was through its anti-angiogenesis activity. Mechanically, we identified the phosphoglycerate mutase 1 (*PGAM1*) gene as a target of sja-miR-61 and found that the sja-miR-61-mediated suppression of cell migration and anti-angiogenesis by cross-species down-regulation of PGAM1 expression. These data indicated that sja-miR-61 is a tumor suppressor miRNA that may have therapeutic potential for human cancers.

## Introduction

MicroRNAs (MiRNAs) is a class of highly conserved small non-coding RNAs and play critical roles in regulation of gene expression *via* binding to their target mRNAs (1). Studies have shown that dysregulated expression of some miRNAs are involved in the occurrence and development of a number of diseases such as cancers (2, 3). These miRNAs may serve as targets for therapeutic intervention (4, 5). Interestingly, recent studies have shown that miRNAs derived from parasites and plants can regulate the expression of mammalian target genes in a cross-species or even a cross-kingdom manner, and thereby affecting the occurrence and development of some human diseases (6–9). For example, the plant-derived miR-159 suppressed the growth of breast cancer cell *via* cross-kingdom regulation of the human transcription factor 7 (*TCF7*) gene (8). Importantly, abundance of miR-159 in the serum of patients was inversely correlated with incidence and progression of breast cancer (8).

Schistosomiasis is a neglected tropical parasitic disease that affects approximately 200 million people in the world’s tropical areas (10). For *Schistosoma japonicum* (*S. japonicum*) infection, numerous eggs laid by the female adult worms were trapped in both liver and intestinal wall tissues, leading to a granulomatous reaction and hepatic fibrosis. Our previous study demonstrated that *S. japonicum* eggs can secrete exosomes that contain *S. japonicum* miRNAs (sja-miRNAs) (11). In addition, sja-miRNAs can be detected in the infected liver hepatocytes and hepatic stellate cells, indicating that sja-miRNAs can be taken up by the host liver cells during the infection (11, 12). We showed that a schistosome-derived sja-miR-2162 that is present in host hepatic stellate cells of infected mice can upregulate collagens and α-SMA production to promote hepatic fibrosis by targeting transforming growth factor beta receptor III (12). In addition, we reported two schistosome-derived miRNAs, sja-miR-3096 and sja-miR-7-5p, that were present in hepatocytes of mice infected with *S. japonicum* inhibited the growth of hepatoma cells by targeting host genes (13, 14). In the present study, we identified an additional *S. japonicum*-specific miRNA-61 (designated as sja-miR-61), which is enriched in extracellular vesicles secreted by *S. japonicum* (EVs) (11), is also present in hepatocytes during the *S. japonicum* infection. This schistosome miRNA suppressed tumor cell migration *in vitro* and growth of hepatoma *in vivo* through anti-angiogenesis by targeting *PGAM1* gene.

## Materials and Methods

### Cell Culture

Both the Hepa1-6 and HepG2 cell lines were grown in Dulbecco’s modified Eagle’s medium (DMEM, Life Technologies, USA) supplemented with 10% (v/v) fetal bovine serum (Invitrogen, USA), 100 U/ml penicillin, and 100 μg/ml streptomycin (Invitrogen, USA) at 37°C in a 5% CO2 incubator, while the HUVEC cell line was grown in Endothelial Cell Medium (ECM, ScienCell, USA), supplemented with 10% (v/v) fetal bovine serum (Invitrogen, USA), 1× Endothelial Cell Growth Supplement (ECGS, ScienCell, USA), 100 U/ml penicillin, and 100 μg/ml streptomycin (Invitrogen, USA) at 37°C in a 5% CO2 incubator.

### Transfection of MiRNA Mimics and Small Interfering RNA

For mimics and small interfering RNA (siRNAs) transfection, exponential growing cells were seeded in the culture plate overnight and transfected with 60 nM sja-miR-61 mimics, siRNAs or negative control mimics (NC; a negative control mimic that has no target gene in human and mice) (Genepharma, Shanghai, China) using Lipofectamine 3000 (Invitrogen, Carlsbad, CA, USA), sja-miR-61mimics: sense 5’-UGA CUA GAA AGU GCA CUC ACU U-3’; anti-sense 5’-GUG AGU GCA CUU UCU AGU CAU U-3’. Human PGAM1 siRNA-1: sense 5’-GUC CUG UCC AAG UGU AUC UTT-3’; anti-sense 5’-AGA UAC ACU UGG ACA GGA CTT-3’. Human PGAM1 siRNA-2: sense 5’-CCA CAU CUG UAG ACA UCU UTT-3’; anti-sense 5’- AAG AUG UCU ACA GAU GUG GTT-3’. Human FERMT2 siRNA: sense 5’-CUG GUG GAG AAA CUC GAU GUA TT-3’; anti-sense 5’-UAC AUC GAG UUU CUC CAC CAG TT-3’. Murine Pgam1 siRNA-1: sense 5’-CCC UAG AAG GUU GGG AUC ATT-3’; anti-sense 5’-UGA UCC CAA CCU UCU AGG GTT-3’. Murine Pgam1 siRNA-2: sense 5’-CGC CUC AAU GAG CGA CAC TTT-3’; anti-sense 5’-AGU GUC GCU CAU UGA GGC GTT-3’. Murine Fermt2 siRNA: sense 5’-UUG GUG GAA AAA CUC GAU GUC TT-3’; anti-sense 5’-GAC AUC GAG UUU UUC CAC CAA TT-3’. NC mimics or NC siRNA: sense 5’-UUC UCC GAA CGU GUC ACG UTT-3’; anti-sense 5’-ACG UGA CAC GUU CGG AGA ATT-3’. For plasmids transfection, exponential growing cells were seeded in the culture plate overnight and transfected with plasmids using Lipofectamine 3000 (Invitrogen, Carlsbad, CA, USA). The cells were incubated for the period of the indicated time, and then subjected to further analysis as described below.

### *In Vitro* Migration Assay

Cell migration was measured by both the Transwell migration and wound healing assay. For Transwell migration assay, Hepa1-6 or HepG2 cells (2×10^5^) and HUVEC (5×10^4^) were seeded in a 6-well plate overnight, then cells were transfected with sja-miR-61 mimics, NC mimics or Mock control (transfection reagents only), respectively. And 24 h later, cells were digested and 2×10^4^ Hepa1-6 or HepG2 cells, 1×10^4^ HUVEC cells were transferred into the upper chamber in 100 μL of serum-free medium, and 500 μL compete medium with 10% (v/v) fetal bovine serum was added to the lower chamber, three replicates per group. And 24 h later, the upper chambers were fixed in methanol for 30 minutes, followed by staining in crystal violet for 15 minutes. After stained, cells on the upper surface of the membrane were wiped out lightly by using the small cotton ball, cells on the lower surface of the membrane were photographed and counted under a light microscope in five fields. For wound healing assay, cells (3×10^5^) cells were seeded in a 6-well plate overnight and then transfected with miRNA mimics as above description. Once confluent, cells were scratched in a straight line using a 200 μL sterile pipette tip. Then, suspended cells were washed off with PBS and cultured in DMEM with 1% (v/v) fetal bovine serum culture. The scratched area was photographed at 0 and 48 h, respectively. The relative area of migration formula = A/B (Where A is the area of migrated cells in experimental group after 48 h; where B is the area of migrated cells in control group after 48 h). The area of migrated cells is evaluated by the ImageJ 1.42q (ImageJ software, Way Rasband, National Institutes of Health, USA).

### Luciferase Reporter Assay

The 3’ untranslated region (UTR) wild-type (WT) and mutant (MT) of *PGAM1* were amplificated from mouse genome or human genome, and then cloned into the Sac I/Xba I site of pmirGLO Dual-Luciferase miRNA Target Expression Vector (Promega, USA), including human pmirGLO-*PGAM1*-WT and mouse pmirGLO-*Pgam1*-WT (containing a wild type binding site in the 3’ UTR of *PGAM1*), human pmirGLO-*PGAM1*-MT and mouse pmirGLO-*Pgam1*-MT (containing a mutant type binding site), and these luciferase reporters were simultaneously transfected with sja-miR-61 mimics or NC mimics in Hepa1-6 or HepG2 cells, respectively. Dual-luciferase reporter assay system (Promega, USA) was used to measure the activity of the reporter gene according to the manufacturer’s instructions, and the firefly luciferase activity was normalized to renilla luciferase activity.

### Mice and *S. japonicum* Infections

All the animal experiments were performed in accordance with the Guide for the Care and Use of Laboratory Animals of the National Institutes of Health, and approved by the Internal Review Board of Tongji University School of Medicine. All the animal surgeries were undertaken under sodium pentobarbital anesthesia. Thirty-six male C57BL/6J mice (6 weeks old, 18-20 g) were purchased from experimental animal center of the Second Military Medical University and housed under specific pathogen-free conditions. Cercariae of *S. japonicum* were provided by National Institute of Parasitic Disease, Chinese Center for Disease Control and Prevention (CDC). Mice were percutaneously infected with cercariae of *S. japonicum*, 50 cercariae for collection of infected hepatocytes and 100 cercariae for collection of early stage parasites (3 mice per group). For collection of parasites, the *S. japonicum* were isolated from the portal system and mesenteric veins of infected mice at 7, 14 and 42 days post-infection (dpi). In addition, at 42 days, female and male adult worms were manually separated. All the freshly isolated parasites were washed with PBS and immediately used for extraction of total RNA or frozen at -80°C until used.

### PGAM1 Overexpression Plasmid Construction

The coding sequence of murine *Pgam1* gene and human *PGAM1* gene were amplified in mouse cDNA or human cDNA, respectively, and cloned into pcDNA3.1(+) vector (Invitrogen, Carlsbad, CA, USA), and the empty pcDNA3.1 (+) vector was used as a control. The primer sequences amplified the *PGAM1* coding sequence are listed in **Table S1**.

### Hepatocellular Carcinoma Xenografts

Six male athymic nude mice (6 weeks old) were housed and manipulated according to the protocols approved by the Shanghai Medical Experimental Animal Care Commission. Hepa1-6 cells or HepG2 cells were transfected with sja-miR-61 mimics or NC mimics at a final concentration of 60 nM, respectively. And 24 h later, 1×10^6^ cells in 100 μL PBS after treated with sja-miR-61 mimics or NC mimics were implanted subcutaneously to per scapula of the nude mice, respectively. The tumor volume was measured every two days after injection. After the last measure, the nude mice were sacrificed and the tumors were separated to evaluate their weight and volume. The tumor volume was determined using the formula: 0.5×L×S^2^, where L or S are the longest or shortest diameter of tumor, respectively. The level of sja-miR-61 mimics transfected into the hepatoma cells were detected by quantitative real-time polymerase chain reaction (qRT-PCR), the expression of Ki67 and CD34 in the tumor was measured by immunohistochemistry as described under this section.

### Western Blot Analysis

The Western blot analysis was performed as described previously (15). Briefly, the cell and tissue sample lysates, extracted by cell lysis buffer (Beyotime, China) and the concentration was measured by Enhanced BCA Protein Assay Kit (Beyotime, China) according to the manufacturer’s instructions. About 20 μg of protein was separated by 12% SDS-PAGE and transferred to a nitrocellulose membrane, respectively. Then the membrane was blocked with 5% bovine serum albumin (BSA) in Tris-buffered saline with Tween (TBST) for 2 h at room temperature, followed by incubated overnight with primary antibodies against PGAM1 (1:500 dilution, Proteintech, China), GAPDH (1:1000 dilution, Beyotime, China). After incubating with the first antibody, the membrane was washed three times with TBST, and then incubated with the relevant secondary antibodies (1:6000 dilution, Promega, USA) for 1 h at room temperature and followed by three times washes, then visualized by using the ECL reagent (GE Healthcare, UK), the protein bands were subsequently measured using the ImageQuant LAS 4000mini (GE Healthcare, USA) and grayscale analysis using the ImageJ 1.42q (ImageJ software, Way Rasband, National Institutes of Health, USA).

### Immunohistochemistry

To determine Ki67, CD34 expression in xenograft tumor tissues from the athymic nude mice, immunohistochemistry (IHC) was performed as described previously (16), Antibody against Ki67, CD34 was used (1:50 dilution). Three mice from each group were used for IHC, and three sections of each tumor were used for analyzing the expression of Ki67 and CD34. For quantification, all IHC photographs were analyzed by using the ImageJ 1.42q (ImageJ software, Way Rasband, National Institutes of Health, USA), following the ImageJ User Guide. Differences in Ki67 and CD34 staining were evaluated by assessing the relative area of positive zone.

### Statistical Analysis

All experiments were performed in triplicate and the results were presented as mean ± standard deviation (mean ± SD). All data were analyzed by one-way ANOVA using the software GraphPad Prism 5.0 (GraphPad Software, Inc. La Jolla, CA, USA). A value of *P* < 0.05 was considered statistically significant.

## Results

### Presence of Sja-miR-61 in Host Hepatocytes of Infected Mice

We first evaluated the presence of sja-miR-61 in the liver cells of infected mice. The sja-miR-61 is a *Schistosoma*-specific miRNA, which allowed us to design the specific primers for detection of this schistosome sja-miR-61 in the hepatocytes of mice infected with *S. japonicum* (**Table S1**). To exclude any contamination from parasite RNA, we carefully prepared the samples of liver cells from the infected mice and showed that a parasite reference gene (*NADH*) with high expression was not detectable in the samples (**Figure S1A**). Then we detected the various time-point samples for presence of sja-miR-61 by qRT-PCR. As shown in **Figure S1B**, the sja-miR-61 was detected at a higher level in the liver cells from early-infected mice (i.e., days 9 and 14 post infection) and the late-infected mice (day 42 post infection) compared with the rest time-point samples. The presence of sja-miR-61 was further verified by PCR as shown in the agarose gel (**Figure S1C**). Furthermore, the PCR product showed identical sequence of sja-miR-61 by cloning and sequencing (**Figure S1D**). These data indicated that sja-miR-61 is present in the host hepatocytes during *S. japonicum* infection.

### Inhibition of Migration of Hepatoma Cells by Sja-miR-61

**Figure 1:**
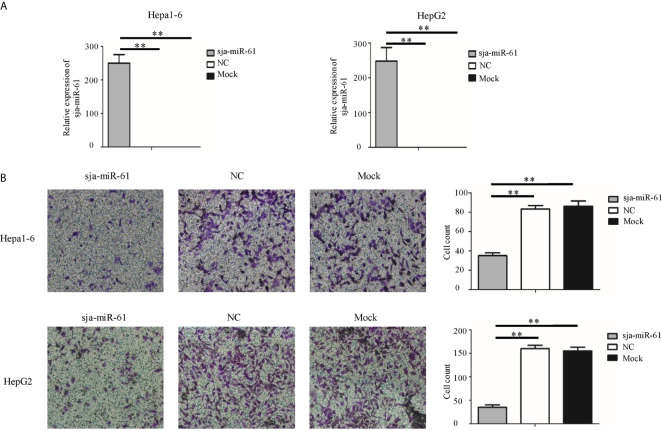
Sja-miR-61-mediated inhibition of migration of hepatoma cells *in vitro*. Hepa1-6 and HepG2 cells were transfected with sja-miR-61 mimics and NC (negative control). mimics, respectively, and 48 h later, the sja-miR-61 was detected by qRT-PCR **(A)**. Cell migration was evaluated using transwell inserts without matrigel coating **(B)**. Data are presented as the mean ± SD, n = 3, **p < 0.01.

To investigate the anti-tumor effects of sja-miR-61 on hepatoma cells *in vitro*, both mouse hepatoma cell lines (Hepa1-6) and human hepatoma cell lines (HepG2) were transfected with the sja-miR-61 mimics, respectively. We showed that the transfected sja-miR-61 mimics were present in the both cell lines (**Figure 1A**), and consequently, significantly inhibited the cell migration of the hepatoma cell lines detected by the transwell migration assay (**Figure 1B**) and the wound healing assay (**Figures S2A, B**) compared with the NC or Mock group. However, we did not observe any inhibitory effect of the sja-miR-61 on the cell cycle and no effect on apoptosis of the hepatoma cell lines (**Figures S3A, B**). These data indicated that sja-miR-61 inhibited the cell migration of both mouse and human hepatoma cells *in vitro*.

### Antitumor Effects of Sja-miR-61 on Hepatoma *In Vivo*

**Figure 2:**
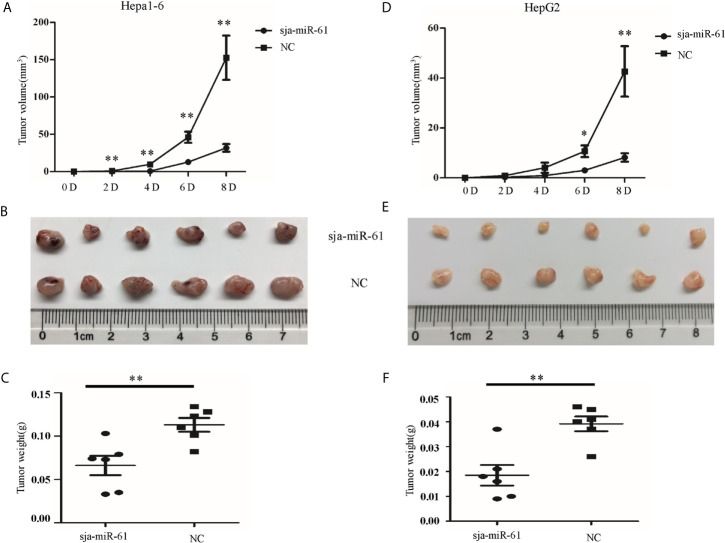
Sja-miR-61-mediated suppression of hepatoma growth *in vivo*. Hepa1-6 and HepG2 cells were transfected with sja-miR-61 mimics or NC mimics, respectively, and then the sja-miR-61-transfected cells (1×10^6^) were injected subcutaneously to the left scapula of athymic nude mice, and the NC-transfected cells to the right scapula (n = 6), respectively. Tumor volumes were measured at days 2, 4, 6, and 8 after injection. At day 8, the mice were sacrificed and tumors were separated to measure their weight and volume, **(A–C)** for Hepa1-6 cells, **(D–F)** for HepG2 cells. Data are presented as the mean ± SD, n = 6, *p < 0.05, **p < 0.01.

To evaluate potential effect of sja-miR-61 on hepatoma *in vivo*, both Hepa1-6 and HepG2 cells were transfected with the sja-miR-61 mimics or NC mimics and then injected subcutaneously into the athymic nude mice respectively to generate subcutaneous tumors. The tumor volume and weight were evaluated at days 2, 4, 6, and 8 post injection. As shown in **Figures 2A–C**, significant reductions in both the tumor volume and weight were observed in the mice inoculated with Hepa1-6 cells transfected with sja-miR-61 mimics compared to those with NC miRNAs. In addition, the similar results were obtained in human hepatoma cell line of HepG2 (**Figures 2D–F**). These data suggested that sja-miR-61 inhibited tumor growth *in vivo*.

### Target Gene of Sja-miR-61

To identify the target gene of sja-miR-61, we used the online software miRDB (17), RNA hybrid (18) and MR-microT (19) to search for putative targets of this miRNA. We found that the seed sequence of sja-miR-61 perfectly binds to the 3’ untranslated region (UTR) of the phosphoglycerate mutase 1 (*PGAM1*) gene of both murine and human. In addition, the *PGAM1* gene was reported as an oncogene during tumorigenesis in humans (20–22).

**Figure 3:**
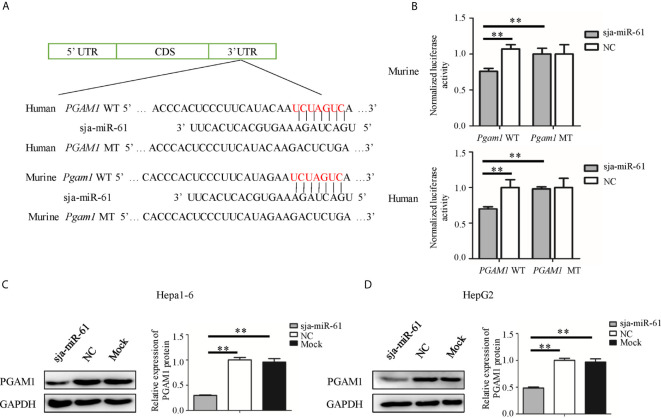
*PGAM1* as target of sja-miR-61. **(A)** A schematic diagram representing the wild-type or mutant 3’ untranslated region (UTR) targeting sites of murine *Pgam1* and human *PGAM1* genes. **(B)** A dual-luciferase reporter assay was used to measure the activity of the reporter gene, and the firefly luciferase activity was normalized to renilla luciferase activity. **(C, D)** The protein levels of murine PGAM1 **(C)** and human PGAM1 **(D)** were measured using Western blotting in the hepatoma cells transfected with sja-miR-61 mimics or NC mimics, respectively. Data are presented as the mean ± SD, n = 3, **p < 0.01.

To verify the *PGAM1* gene as the target of sja-miR-61, we constructed several plasmids that expressing the luciferase reporter, in which the firefly luciferase gene is fused to the 3’ UTR of *PGAM1* gene of human (pmirGLO-*PGAM1*-WT) and murine (pmirGLO-*Pgam1*-WT), as well as their mutants (pmirGLO-*PGAM1*-MT and pmirGLO-*Pgam1*-MT, respectively) where the seven nucleotides of the binding site were mutated (**Figure 3A**). Both Hepa1-6 and HepG2 cells were simultaneously transfected with the relevant plasmids and sja-miR-61 mimics or NC mimics. As shown in **Figure 3B**, a significant reduction of the luciferase activity was detected in the Hepa1-6 cells transfected with the pmirGLO-*Pgam1*-WT but not with the pmirGLO-*Pgam1*-MT. Similar results were obtained with the human cell line of HepG2 (**Figure 3B**). These results suggested that sja-miR-61 directly bind to the 3’ UTR of the *PGAM1* gene, but not its mutant, to down-regulate the expression of luciferase reporter gene.

We then evaluated the expression of the PGAM1 protein in both Hepa1-6 and HepG2 cells by Western blotting after transfected with sja-miR-61 mimics. The results showed that sja-miR-61 significantly down-regulated the levels of PGAM1 in hepatoma cells compared to the NC or Mock controls (**Figures 3C, D**).

### Sja-miR-61-Mediated Inhibition of the Cell Migration by Targeting PGAM1 *In Vitro*

**Figure 4:**
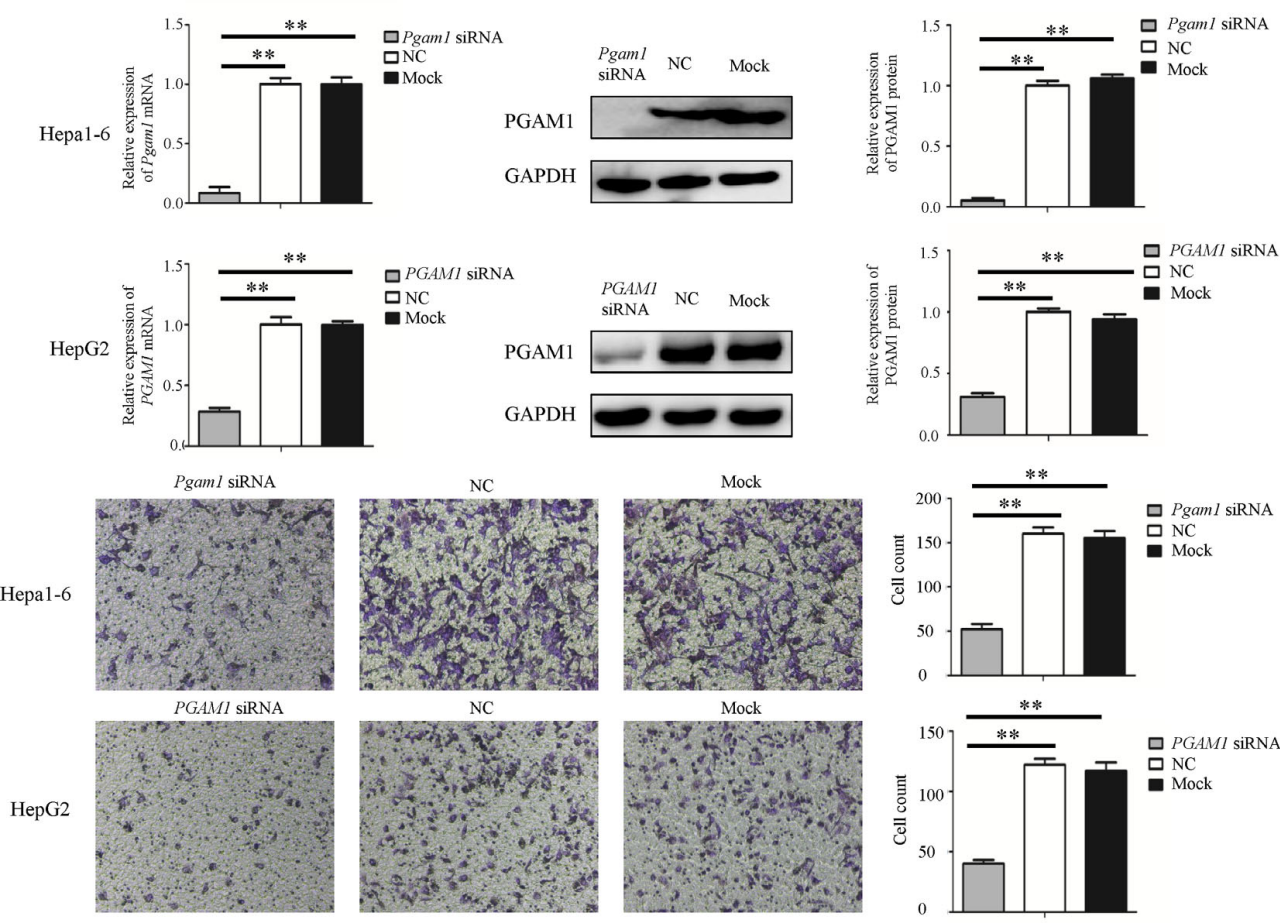
Knockdown of *PGAM1* inhibits cell migration of hepa1-6 and HepG2 cells *in vitro.* **(A, B)** Hepa1-6 and HepG2 cells were transfected with *PGAM1* siRNA and negative control (NC) siRNA, respectively, and 48 h later, the expression of *PGAM1* was determined using qRT-PCR and Western blotting **(A)**. Cell migration was evaluated using transwell inserts without matrigel coating **(B)**. Data are presented as the mean ± SD, n = 3, **p < 0.01.

To investigate whether sja-miR-61 inhibits the migration of hepatoma cells by targeting *PGAM1*, both Hepa1-6 and HepG2 cells were transiently transfected with the *PGAM1* siRNAs, NC or Mock controls. We found that both murine *Pgam1* siRNA and human *PGAM1* siRNA obviously reduced the *PGAM1* expression in Hepa1-6 cells and HepG2 cells, as measured by qRT-PCR and Western blotting, respectively (**Figure 4A**). Importantly, similar to the findings in the sja-miR-61 mimics-transfected cells, the siRNAs treatment led to inhibition of migration of both Hepa1-6 and HepG2 cells compared to the NC- or Mock-treatments (**Figure 4B**).

**Figure 5:**
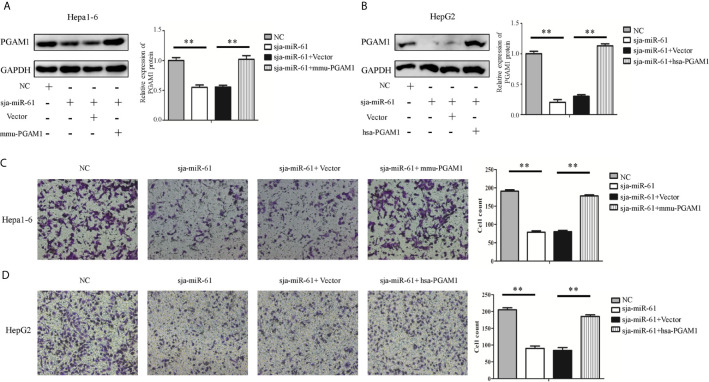
Effect of restoration of PGAM1 expression on the sja-miR-61-mediated effects *in vitro*. **(A–D)** Cells were transfected with NC mimics, sja-miR-61 mimics, NC mimics + pcDNA3.1(+) vector, sja-miR-61 mimics + pcDNA3.1(+)-PGAM1, respectively, and 48 h later, the expression of PGAM1 was determined using Western blotting **(A, B)**. Cell migration was evaluated using transwell inserts without matrigel coating **(C, D)**. Mean ± SD, n = 3, **p < 0.01.

We next transfected both Hepa1-6 and HepG2 cells with the constructs expressing the murine *Pgam1* or human *PGAM1* genes respectively that lack its 3’ UTR so that sja-miR-61 should not affect the expression of the transfected PGAM1 gene. As shown in **Figures 5A, B**, the sja-miR-61 significantly reduced the expression of endogenous *PGAM1* gene, but not the transfected gene in both cell lines, as the expression of PGAM1 returned to the original level when transfected with the *PGAM1*-expressing constructs compared to the vector control. Importantly, restoration of PGAM1 level led to abolishment of the sja-miR-61-mediated inhibition of the migration of the hepatoma cell in both cell lines (**Figures 5C, D**). These results indicated that the inhibitory effects of the sja-miR-61 on migration of the hepatoma cells by down-regulation of the *PGAM1* expression.

### Sja-miR-61-Mediated Suppression of the Hepatoma Growth *In Vivo* Through Anti-Angiogenesis by Targeting PGAM1 Gene

**Figure 6:**
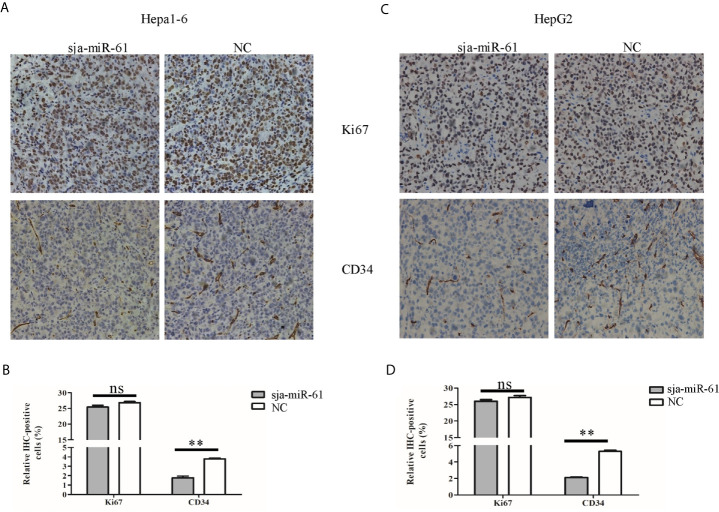
Detection of the expression of both Ki67 and CD34 in tumors. **(A–D)** The expression levels of both Ki67 and CD34 in tumors were determined using immunohistochemistry, **(A, B)** for Hepa1-6 cells, **(C, D)** for HepG2 cells. Data are presented as the mean ± SD, n = 6, **p < 0.01, ns indicates no significant.

As described above, sja-miR-61 has no inhibitory effect on growth of both Hepa1-6 and HepG2 cells *in vitro* (**Figures S3A, B**), but significantly suppressed growth of tumor cells *in vivo* (**Figures 2A–F**). To explore the potential molecular mechanism by which sja-miR-61 inhibited *in vivo* growth of hepatoma, we detected the expression levels of both Ki67 and CD34 using immunohistochemistry (IHC). CD34 is an angiogenesis marker and expressed in certain type of cells including capillary endothelial cells. We showed no change in the expression of Ki67, indicating no impact on the cell proliferation, similar to the data from *in vitro* experiments. However, the expression of CD34 was significantly decreased in tumor tissues of both Hepa1-6 and HepG2 cells transfected with the sja-miR-61 mimics compared with the NC control (**Figures 6A–D**), suggesting the inhibitory effect of sja-miR-61 on angiogenesis in the tumor.

**Figure 7:**
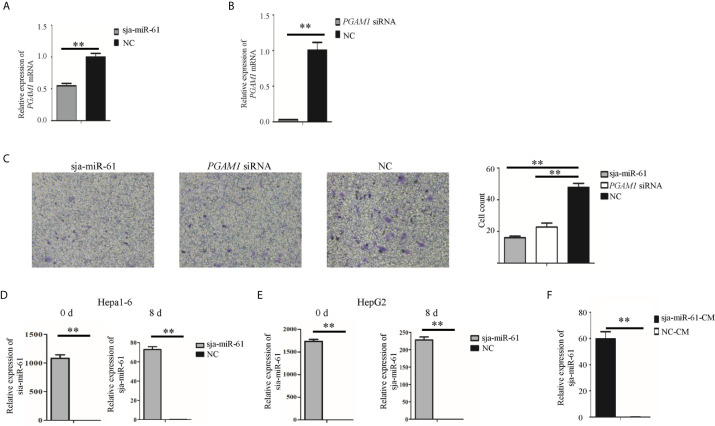
Sja-miR-61-mediated inhibition of migration and tube formation of HUVEC cells by down-regulating *PGAM1 in vitro*. **(A, B)** HUVEC cells were transfected with sja-miR-61 mimics and NC mimics **(A)**, *PGAM1* siRNA and NC siRNA **(B)**, respectively, and 48 h later, the expression of *PGAM1* was determined using qRT-PCR. Cell migration was evaluated using transwell inserts without matrigel coating **(C)**. **(D, E)** The sja-miR-61 mimics within the transfected cells were measured by qRT-PCR at day 0 (i.e. before inoculation) and 8 post inoculation, using U6 as the internal control, **(D)** for Hepa1-6 cells, **(E)** for HepG2 cells. **(F)** The sja-miR-61 mimics were detected by qRT-PCR in HUVEC cells cultured with conditioned medium (CM) derived from the cultivation of HepG2 cells transfected with sja-miR-61 mimics or NC mimics. Mean ± SD, n = 3, **p < 0.01.

We next investigate if the sja-miR-61 mediates inhibition of angiogenesis by targeting *PGAM1* gene. Migration of vascular endothelial cells is important factor that affect angiogenesis (23). Thus, we used the human umbilical vein endothelial cells (HUVEC) for the assays of the migration. The HUVEC cells were transfected with the sja-miR-61 mimics and *PGAM1* siRNA, respectively. As shown in **Figures 7A, B**, both sja-miR-61 mimics and *PGAM1* siRNA significantly reduced the *PGAM1* expression in the HUVEC cells detected by qRT-PCR. Importantly, the transwell migration showed that transfection of the HUVEC cells with either sja-miR-61 mimics or *PGAM1* siRNA led to significantly inhibition of the cell migration compared with the NC control, respectively (**Figures 7C**). These data suggested that the inhibitory effects of the sja-miR-61 on the migration of HUVEC could be through down-regulating *PGAM1* expression.

To understand how the sja-miR-61 mimics enter vascular endothelial cells *in vivo*, we first detected the presence of sja-miR-61 in the tumors. We found that the sja-miR-61 was still detectable in the tumors on day 8 after injection of the transfected hepatoma cells with the miRNA, although the level was much lower than that on day 0 (**Figures 7D, E**). We speculated that the transfected sja-miR-61 mimics in the hepatoma cells can be secreted to the extracellular, and then ingested by vascular endothelial cells. To verify this, a conditioned medium (CM) was prepared by cultivation of the HepG2 cells transfected with sja-miR-61 mimics, and then the CM was used for *in vitro* culture of HUVEC cells. We detected and showed the presence of the sja-miR-61 inside the HUVEC cells (**Figure 7F**), suggesting that the sja-miR-61 mimic can be secreted from the transfected hepatoma cells to the medium and then ingested by the endothelial cells.

## Discussion

Accumulated evidences have demonstrated the anti-tumor effects of certain miRNAs through suppression of tumor cell growth, migration and invasion, including those miRNAs derived from plants and parasites. In this study, we showed that the schistosome miRNA, sja-miR-61, is present in host hepatocytes during schistosome infection, has evident inhibition of migration of hepatoma cells *in vitro*. Importantly, this schistosome miRNA exert a notable anti-angiogenesis activity as the expression of the angiogenesis marker (CD34) was significantly decreased in tumor tissues. The inhibition of angiogenesis activity could be through suppression of migration of the vascular endothelial cells which we demonstrated *in vitro* experiments. As to question about the entering of the sja-miR-61 mimics into vascular endothelial cells *in vivo*, we showed that the transfected hepatoma cells with the sja-miR-61 mimics could secrete the miRNA to the culture medium and ingested by the endothelial cells in an *in vitro* model, and that the sja-miR-61 mimics were detectable in the tumors till the 8^th^ day, which imply that *in vivo* the transfected tumor cells may secrete the sja-miR-61 mimics to the extracellular and then enter the vascular endothelial cells to exert anti-angiogenesis. In addition, the analysis of molecular mechanism revealed that sja-miR-61 exerts the inhibitory effects on both cell migration and angiogenesis by targeting the *PGAM1* gene. Thus, the present data indicated that the schistosome sja-miR-61 is a tumor suppressor miRNA that may have therapeutic potential for human cancers.

Infection with certain parasites have been reported to be associated with cancers, such as *Clonorchis sinensis* and *Opisthorchis viverrine* (24, 25). It was also documented that *Schistosoma haematobium* infection is associated with bladder cancer (24, 25). For *S. japonicum* infection, a potential association with colorectal cancer was reported (26), but it is less evident for the association between *S. japonicum* infection and HCC. Previous studies revealed that chronic inflammation was involved in the tumorigenesis (27). As to *S. japonicum* infection, the parasite eggs induce severe chronic inflammation and fibrosis, which should be risk factors for HCC (28). Thus, we speculated that the eggs of *S. japonicum* trapped in the liver may play a dual role in the occurrence and development of HCC, i.e. carcinogenic and anti-cancer activities, as reported in the infection with protozoan *Trypanosoma cruzi* (29). The present study demonstrated that the sja-miR-61 derived from *S. japonicum* had a notable anti-tumor activity. Thus, both the presence of this miRNA, together with the other reported schistosome miRNAs such as sja-miR-3096 (13) and sja-miR-7-5p (14), in host hepatocytes and their antitumor effects on human hepatoma cells may suggest the schistosome miRNA-mediated anti-tumor effects during schistosome infection.

To identify the target gene of the parasite sja-miR-61, we first used three online software to search for its potential target genes. We found 5 potential target genes (*SKP2, SMO, SDCBP, FERMT2 and PGAM1)* that were consistently predicted by the three softwares and involved in tumor-related signaling pathway. The luciferase reporter assay showed the genes of *SMO*, *SDCBP*, *FERMT2* and *PGAM1* as potential targets of the sja-miR-61 (**Figure 3**; **Figures S4A–F**; **Figures S5A, B**). However, the transfection experiments with siRNA or miRNA mimics excluded the *SMO* and *SDCBP* as the targets (**Figures S4G, H**). For both *PGAM1* and *FERMT2* gene, we observed that sja-miR-61 down-regulated their expression, which led to the inhibition of the hepatoma cell migration *in vitro*, similar to that in the cell with their siRNA (**Figure 3** and **Figure S5**), but the *PGAM1-*mdediated inhibition degree was higher than the *FERMT2-mediated* inhibition in terms of their expression levels and the cell migration. Thus, we focused on the *PGAM1* gene as the major target of the sja-miR-61 for next experiments, including the evaluation of the inhibitory effect on migration of vascular endothelial cells, and the restoration of PGAM1 expression. We demonstrated that the restoration of PGAM1 expression rescued the sja-miR-61-mediated anti-tumor effects.

PGAM1 is an important enzyme in glycolysis, which catalyzes the conversion of 3−phosphoglycerate (3−PG) to 2−phosphoglycerate (2−PG) (21). Several studies reported that PGAM1 is overexpressed in various cancers, including hepatocellular carcinoma (30), lung cancer (31), breast cancer (32), prostate cancer (22) and renal clear cell carcinoma (33). *PGAM1* is characterized as an oncogene, and is involved in modulation of the cell proliferation, migration, invasion (20–22). In the present study, our data suggested that *PGAM1* be involved in regulation of angiogenesis. Tumor angiogenesis is essential for tumor growth and metastases. We demonstrated that the sja-miR-61 inhibited migration of vascular endothelial cells *in vitro* through targeting *PGAM1* gene, which consequently affected angiogenesis *in vivo*.

Tumor microenvironment (TME), a complex ecosystem comprising of tumor cells, immune cells, and stromal populations including vascular cells and fibroblasts (34, 35). Our data showed that vascular endothelial cells in tumors could take up sja-miR-61 secreted by tumor cells, which in turn affects the migration of vascular endothelial cells, and thereby hinders tumor growth. However, it is undeniable that the sja-miR-61 treated hepatoma cells might affect their own growth through autocrine, and might also affect other stromal cells through paracrine, which in turn affects the growth of tumor cells. Thus, our study has several limitations: first, it is not clear if the sja-miR-61 treated hepatoma cells can affect the growth of vascular endothelial cells, stroma cells and tumor cells through autocrine and paracrine interactions; Second, our data showed that *in vitro* sja-miR-61 has an inhibitory effect on the migration of hepatoma cells and vascular endothelial cells. However, the effect *in vivo* has not been explored yet, including the effect of sja-miR-61 on tumor metastasis; Third, the effect of sja-miR-61 on clinical tumors is not examined as it is extremely difficult to obtain clinical samples with schistosomiasis.

In conclusion, these data imply that sja-miR-61 might strengthen resistance of host to cancer during schistosome infection, and the discovery and development of such heterogenous anti-tumor miRNAs may provide a novel approach for human cancer therapeutic intervention.

## Data Availability Statement

The original contributions presented in the study are included in the article/**Supplementary Material**. Further inquiries can be directed to the corresponding author.

## Ethics Statement

All the animal experiments were performed in accordance with the Guide for the Care and Use of Laboratory Animals of the National Institutes of Health, and approved by the Internal Review Board of Tongji University School of Medicine.

## Author Contributions

CH and WP conceived and designed the study. CH, YLi, DP, JW, LZ, YLin and SZ performed the experiments. CH, YLi, DP and WP analyzed the data. CH and WP wrote the manuscript. All authors contributed to the article and approved the submitted version.

## Funding

This study was supported by the National Natural Science Foundation of China (81972985).

## Conflict of Interest

The authors declare that the research was conducted in the absence of any commercial or financial relationships that could be construed as a potential conflict of interest.

## Abbreviations

*S. japonicum*, *Schistosoma japonicum*; HCC, hepatocellular cancer; PGAM1, phosphoglycerate mutase 1; miRNA, microRNA; siRNA, small interfering RNA; NC, negative control; Mock, mock control.
