# Supplementary material for: A Schistosoma japonicum MicroRNA Exerts Antitumor Effects Through Inhibition of Both Cell Migration and Angiogenesis by Targeting PGAM1
Source: Front Oncol. 2021 Jun 16;11:652395. doi: 10.3389/fonc.2021.652395 (PMC8242254; doi:10.3389/fonc.2021.652395)
Supplement: Supplementary file 2 [file DataSheet_2.docx]

**Title:**

**A *Schistosoma japonicum* MicroRNA Exerts Antitumor Effects Through Inhibition of Both Cell Migration and Angiogenesis by** **Targeting PGAM1**

**Authors:**

Chao Hu^1^, Yuzhen Li^1^, Danting Pan^1^, Jing Wang^1^, Liufang Zhu^1^, Yu Lin^1^, Shanli Zhu^1^, Weiqing Pan^1,2*^

**Authors' affiliations:**

^1^ Institute for Infectious Diseases and Vaccine Development, Tongji University School of Medicine, Shanghai, China

^2^ Department of Tropical Diseases, Naval Medical University, Shanghai, China

**^*^Corresponding author:**

Weiqing Pan, Ph.D., Department of Tropical Diseases, Naval Medical University, 800 Xiang Yin Road, Shanghai 200433, China; E-mail: wqpan0912@aliyun.com (W.P.)

**SUPPLEMENTARY MATERIALS AND METHODS**

**Cell Cycle Analysis**

Hepa1-6 and HepG2 cells (1×10^5^) were transfected with sja-miR-61 mimics, NC mimics or Mock for 48 h, respectively. Then, cells were collected and fixed with ice-cold 75 % (v/v) ethanol at 4°C overnight. Next, cells were washed and resuspended in 200 μL phosphate-buffered saline (PBS) contained with 25 mg/mL propidium iodide (PI; Beyotime, China) and 0.05 mg/mL RNase A (Beyotime, China), cell cycle was detected by the FACSverse ﬂow cytometer (BD Biosciences, USA). Data were collected and analyzed with FlowJo software.

**Cell Apoptosis Analysis**

Apoptotoc cells were measured by using the Annexin V Apoptosis Dection Kit FITC (eBioscience, USA) according to the manufacturer's instructions. In brief, cells were transfected with sja-miR-61 mimics or NC mimics, respectively, three replicates per group. And 48 h later, cells were collected and washed with PBS, then resuspended in 100 μL 1×binding buffer with 5 μL FITC-conjugated anti-Annexin V antibody. Then, cells were incubated in the dark for 15 min at room temperature. After incubated, cells were washed in 1×binding buffer and resuspend in 200 μL of 1× binding buffer with 5 μL PI. Apoptosis was analyzed by the FACSverse ﬂow cytometer (BD Biosciences, USA).

**Isolation of** **Primary Hepatocytes of** ***S. japonicum* infected Mice**

The primary hepatocytes of *S. japonicum* infected or uninfected mouse were isolated as described by He et al (1) with minor modifications. Briefly, livers from the infected mice were collected at different time points of 7, 9, 11, 14, 28 and 42 dpi (n=5), along with the livers from uninfected mice were initially in situ digested with 0.03% collagenase type IV and further digested with 0.08% collagenase type IV at 37°C for 30 min in a shaking bath. The single hepatocytes were harvested by filtration through 400-mesh sieves to exclude parasite eggs and the remaining tissue debris. Then, cell suspensions were isolated by centrifugation at 50×g for 4 min and further purified by repeated centrifugation. Purified hepatocytes were resuspend in DMEM containing 20 μg/mL Ribonuclease A (Sigma-Aldrich, USA) and incubated at 37°C for 30 min to eliminate any RNA that might be released by parasites. After incubation, the cells were washed by PBS for three times and immediately used for extraction of total RNA or frozen at -80°C until used.

**RNA Preparation, Reverse Transcription, PCR, QRT-PCR**

For RNA extraction, total RNA was extracted from cells, tumor samples or *Schistosoma japonicum* by using Trizol reagent (Life technologies, Carlsbad, CA, USA) according to the manufacturer's instructions, while the RNA of primary mouse hepatocytes from *Schistosoma japonicum* infected mouse was extracted by using miRNeasy Mini kit (Qiagen, Germany) according to the manufacturer's instructions. For RNA reverse transcription, miRNA were reverse transcribed into cDNA using Reverse Transcriptase M-MLV kit (Takara, Dalian, China) according to the manufacturer's instructions, and mRNA were transcribed into cDNA using PrimeScript RT Master Mix reagent kit (Takara, Dalian, China) according to the manufacturer's instructions. For qRT-PCR, which was using SYBR green assay kit (Takara, Dalian, China) with the Applied Biosystems 7500 were performed as described previously (2), U6 and GAPDH were used as internal control for miRNA and mRNA, respectively. The relative expression level of miRNA and mRNA was calculated by 2^-ΔΔCt^ method. All PCR assays were run in triplicate. For ordinary PCR, the amplification procedure was the same as qRT-PCR but only 32 cycles, the amplification products were performed with the 2% agarose gel electrophoresis. All PCR experiments were done in triplicate. All the primers are listed in Table S1.

**SUPPLEMENTARY TABLES AND FIGURES**

| **Table S1**. Sequences of primers used for qRT-PCR or ordinary PCR. | | |
| --- | --- | --- |
| **Gene** | **Name** | **Sequence(5'-3')** |
| sja-miR-61 | Reverse transcription stem-loop- primer RT | CTCAACTGGTGTCGTGGAGTCGGCAATTCAGTTGAGAAGTGAG |
|  | Forward primer FP | ACACTCCAGCTGGGTGACTAGAAAGTG |
|  | Common reverse primer | CTGGTGTCGTGGAGTCGGCAA |
| sja-U6 | Forward primer | CGGCGGTACATATACTAAAAT |
|  | Reverse primer | AACGCTTCACGATTTTGCGT |
|  | Reverse transcription stem-loop- primer RT | TATGGAACGCTTCACGATTTTG |
| mmu-U6 | Forward primer | GCTTCGGCAGCACATATACTAAAAT |
|  | Reverse primer & Reverse transcription stem-loop- primer RT | CGCTTCACGAATTTGCGTGTCAT |
| mmu-*Gapdh* | Forward primer | GTGTTCCTACCCCCAATGTGT |
|  | Reverse primer | GTCATACCAGGAAATGAGCTTGA |
| mmu-*Pgam1* | Forward primer | TCTGTGCAGAAGAGAGCAATCC |
|  | Reverse primer | CTGTCAGACCGCCATAGTGT |
| hsa-*GAPDH* | Forward primer | ACAACTTTGGTATCGTGGAAGG |
|  | Reverse primer | GCCATCACGCCACAGTTTC |
| hsa-*PGAM1* | Forward primer | GTGCAGAAGAGAGCGATCCG |
|  | Reverse primer | CGGTTAGACCCCCATAGTGC |

**
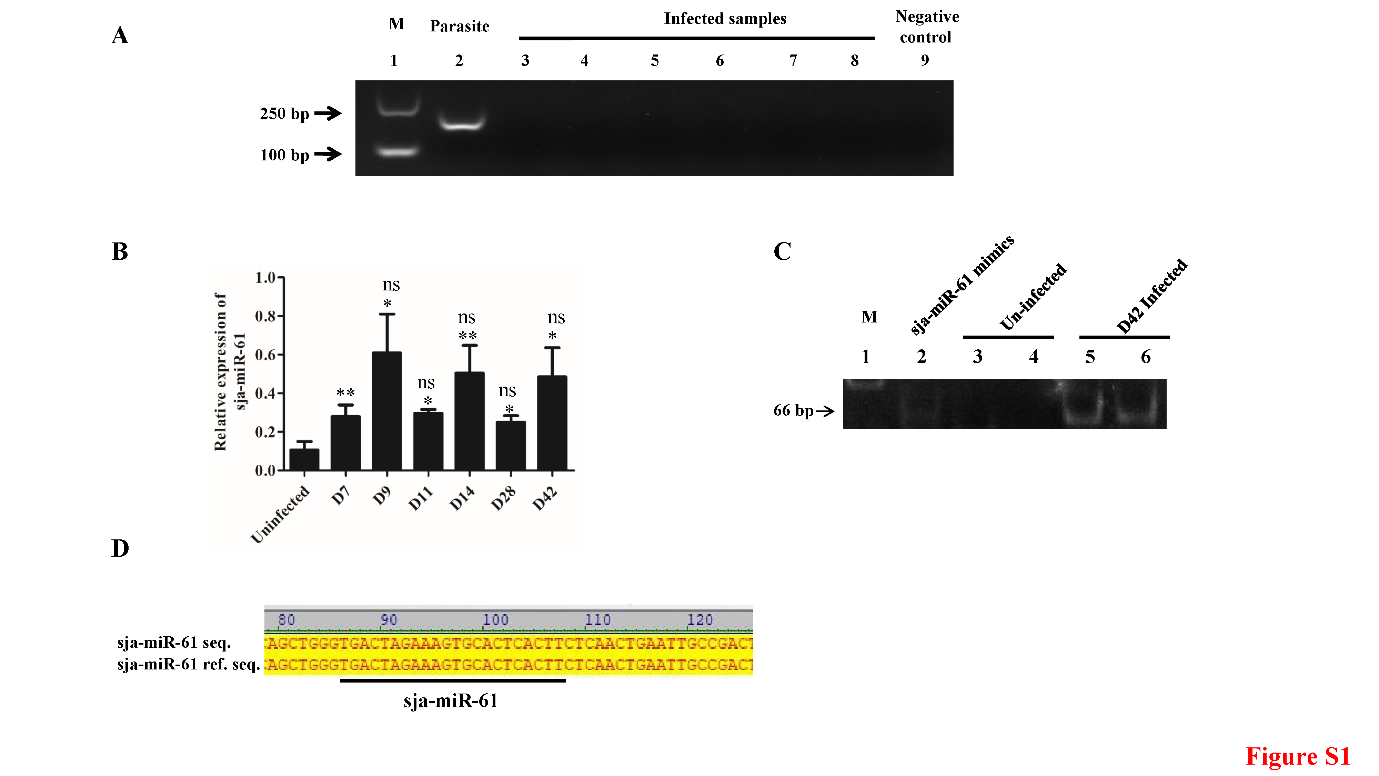
**

**Figure S1. Detection of sja-miR-61 in the infected hepatocytes.** (A) Analysis of the RNA samples from liver cells of infected mice to ensure no contamination with parasite RNA: the RNA samples were detected as described in Method by PCR for presence of the *NADH* gene of *S. japonicum*. Lane 1: marker. Lane 2: parasite positive control: RNA samples of *S. japonicum* eggs as described above. Lane 3-8: six samples of hepatocytes of infected mice with RNase pre-incubation. Lane 9: negative control without the template. (B) qRT-PCR analysis of sja-miR-61 in the hepatocytes of infected mice at various days after infection. Data are presented as the mean ± SD, n = 5, **p* < 0.05 versus the uninfected control group, ***p* < 0.01 versus the uninfected control group, ns indicates no significant versus Day 7 sample group. (C) 12% PAGE analysis showing sja-miR-61 PCR product (66 bp) from the hepatocytes : Lane 1: marker; Lane 2: sja-miR-61 mimics positive control; Lanes 3 and 4: two uninfected hepatocyte samples with pre-incubation with RNase; Lanes 5 and 6: two infected hepatocyte samples at day 42 post-infection with the pre-incubation. (D) The sequence alignment of the amplified sja-miR-61 from the infected hepatocytes and its reference sequence.

**
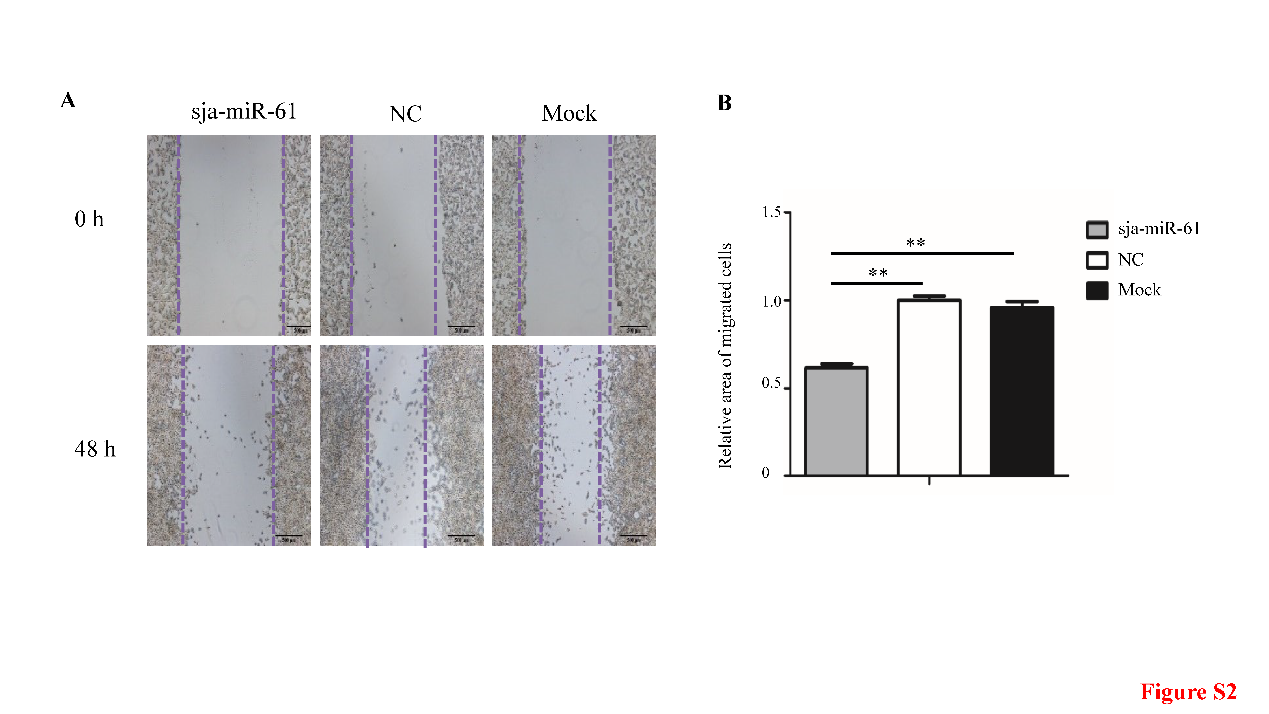
**

**Figure S2. Sja-miR-61 inhibits cell migration of Hepa1-6 cells *in vitro*.** (A,B) Hepa1-6 cells were transfected with sja-miR-61 mimics and negative control (NC) mimics, respectively, and 48 h later, the cell migration was evaluated using wound-healing assay. Data are presented as the mean ± SD, n = 3, ***p* < 0.01.

**
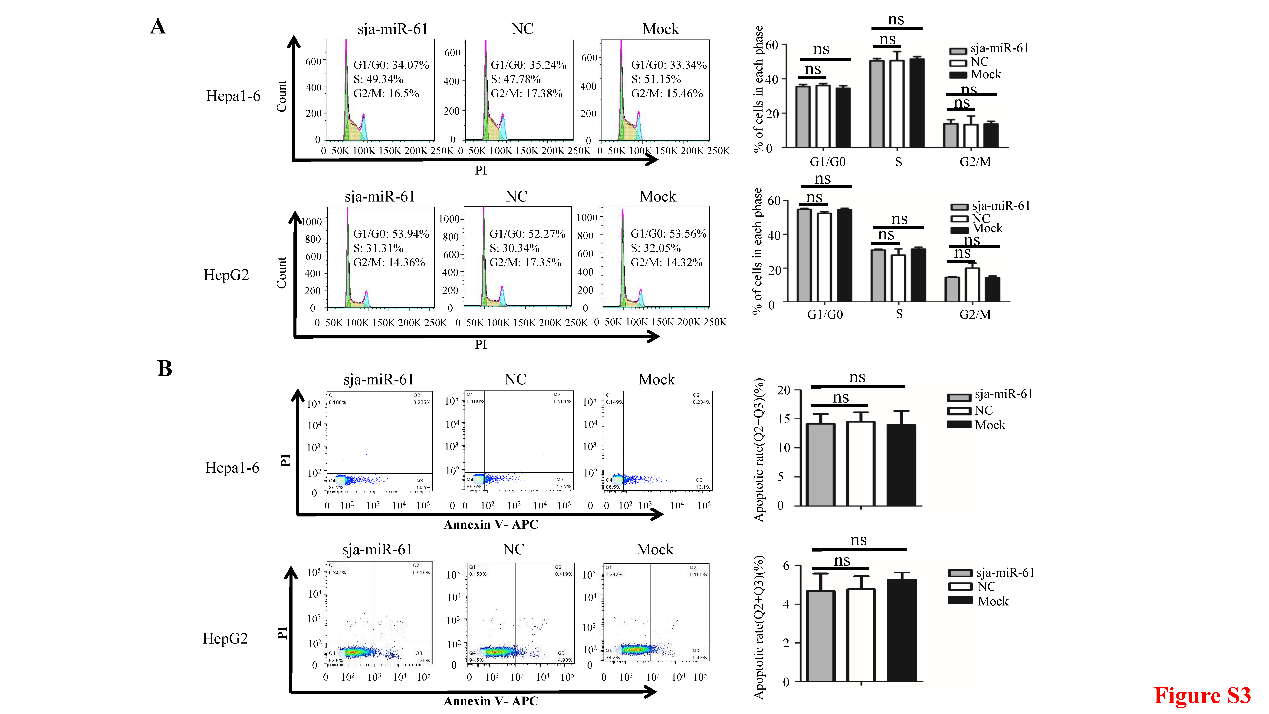
**

**Figure S3. Effect of sja-miR-61 on cell cycle and apoptosis of hepatoma cells *in vitro*.** (A-B) Hepa1-6 and HepG2 cells were transfected with sja-miR-61 mimics and NC (negative control) mimics, respectively, and 48 h later, the cell cycle (A) and cell apoptosis (B) were determined by flow cytometry analysis. Data are presented as the mean ± SD, n = 3, ns indicates no significant.


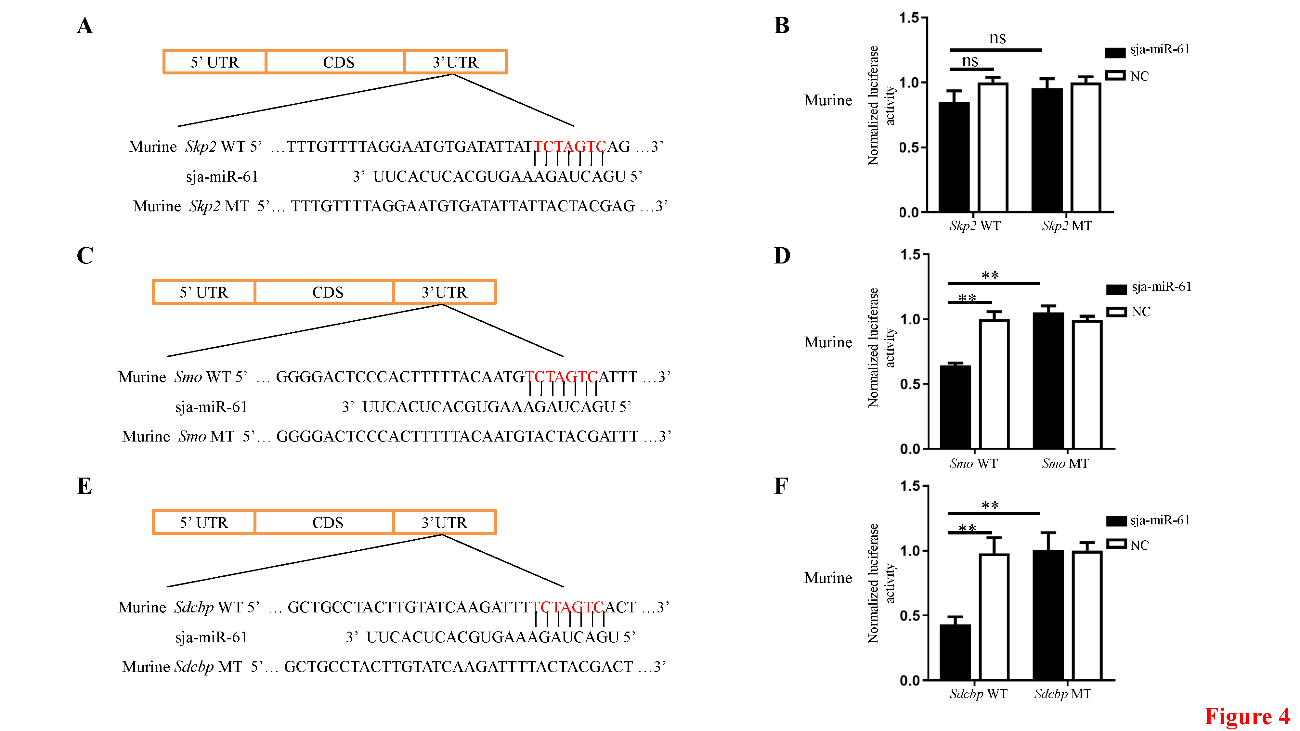


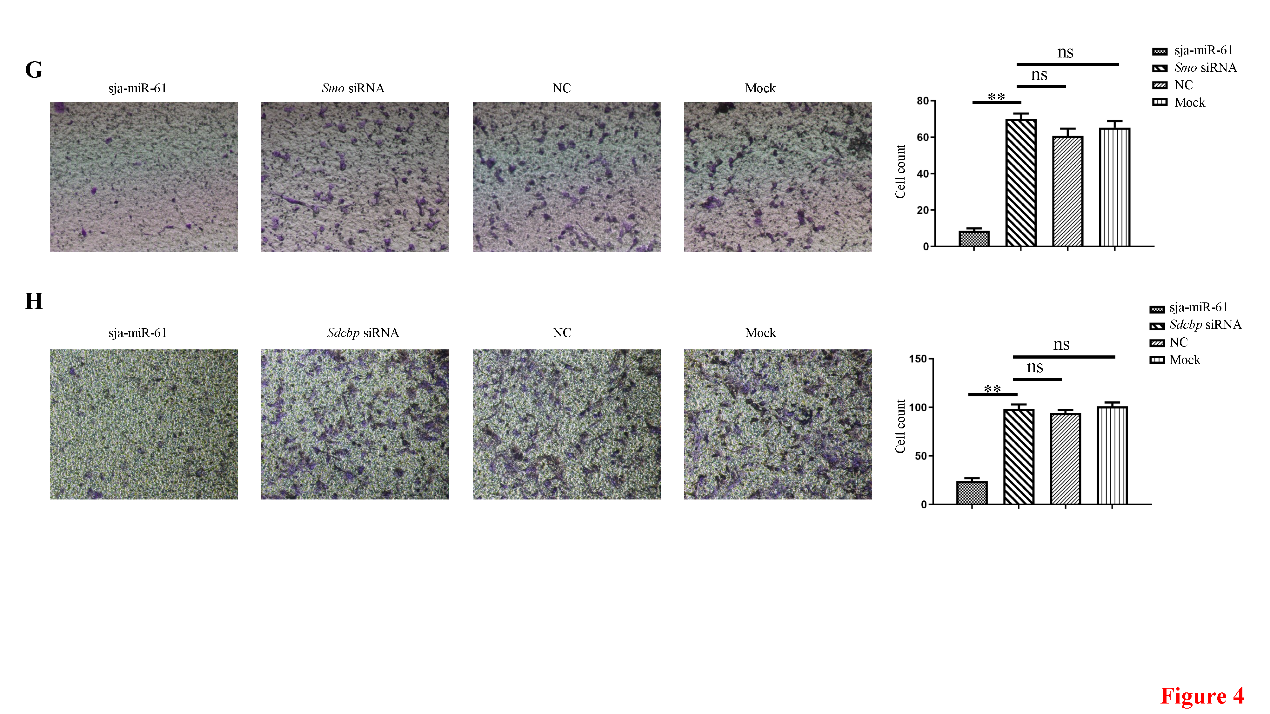


**Figure S4. Screening of the potential target genes of sja-miR-61.** (A,C,E) A schematic diagram representing the wild-type or mutant 3' untranslated targeting region (UTR) sites of murine *Skp2* (A)*, Smo* (C)*, Sdcbp* gene (E). (B,D,F) A dual-luciferase reporter assay was used to measure the activity of the reporter gene, and the firefly luciferase activity was normalized to renilla luciferase activity. B for *Skp2* gene*,* D for *Smo* gene*,* F for *Sdcbp* gene. (G,H) Hepa1-6 cells were transfected with sja-miR-61 mimics, *Smo* (G) or *Sdcbp* siRNA (H) and negative control (NC) siRNA, respectively, and 48 h later, cell migration was evaluated using transwell inserts without matrigel coating. Data are presented as the mean ± SD, n = 3, ***p* < 0.01, ns indicates no significant.

**
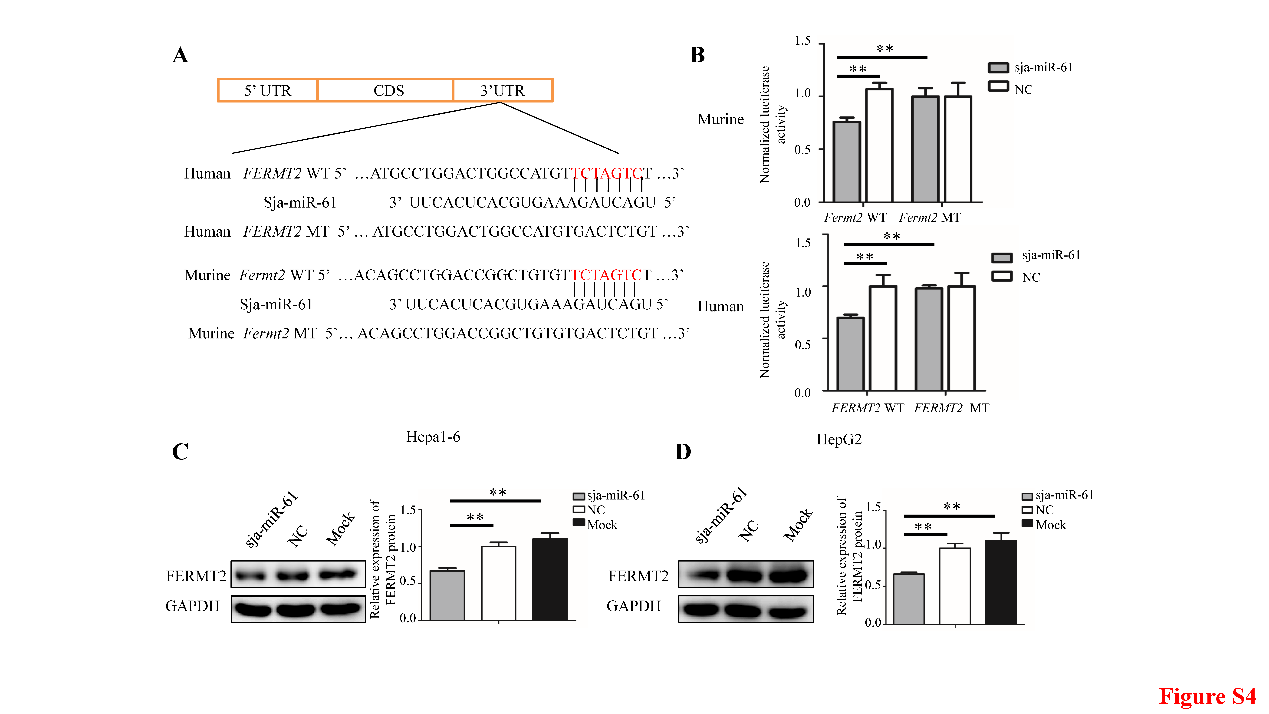
**

**
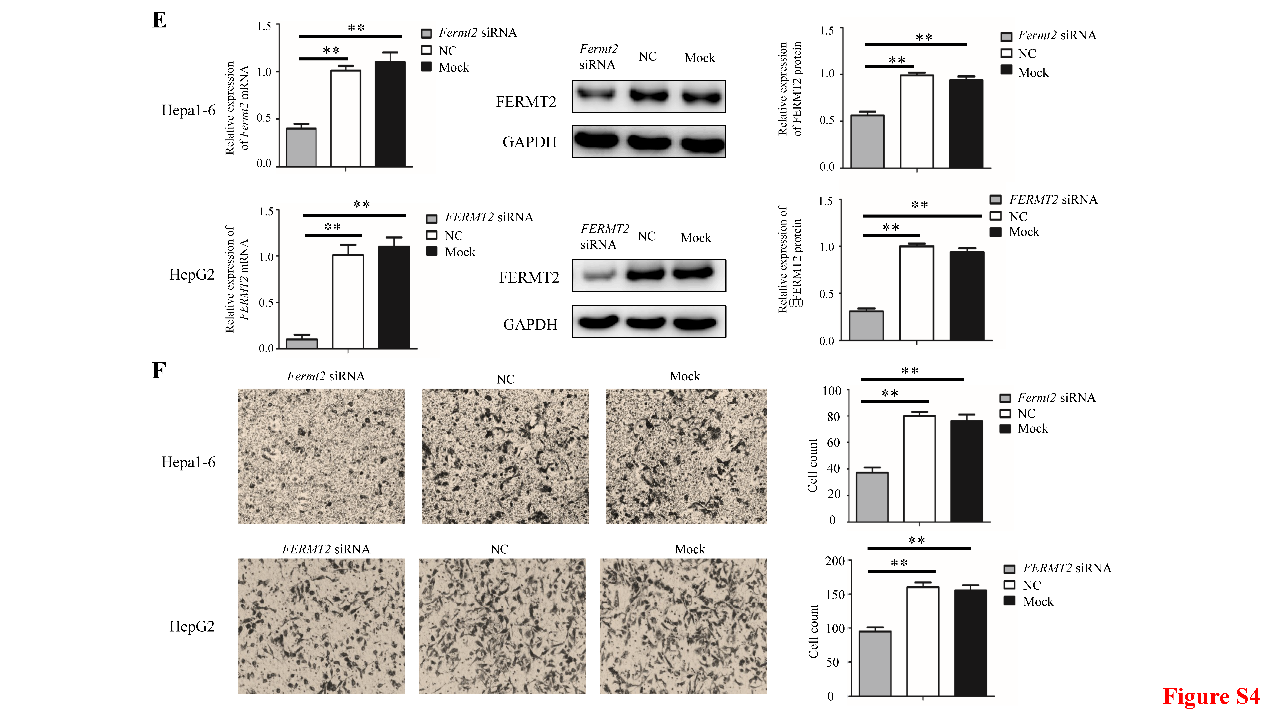
**

**Figure S5. Evaluation of *FERMT2* as a potential target of sja-miR-61.** (A) A schematic diagram representing the wild-type or mutant 3' untranslated targeting region (UTR) sites of murine *Fermt2* and human *FERMT2* genes. (B) A dual-luciferase reporter assay was used to measure the activity of the reporter gene, and the firefly luciferase activity was normalized to renilla luciferase activity. (C,D) The protein levels of murine FERMT2 (C) and human FERMT2 (D) were measured by Western blotting in the cells transfected with sja-miR-61 mimics or NC mimics, respectively. (E,F) Hepa1-6 and HepG2 cells were transfected with *FERMT2* siRNA and negative control (NC) siRNA, respectively, and 48 h later, the expression of *FERMT2* was determined using qRT-PCR and Western blotting (E). Cell migration was evaluated using transwell inserts without matrigel coating (F). Data are presented as the mean ± SD, n = 3, ***p* < 0.01.

**
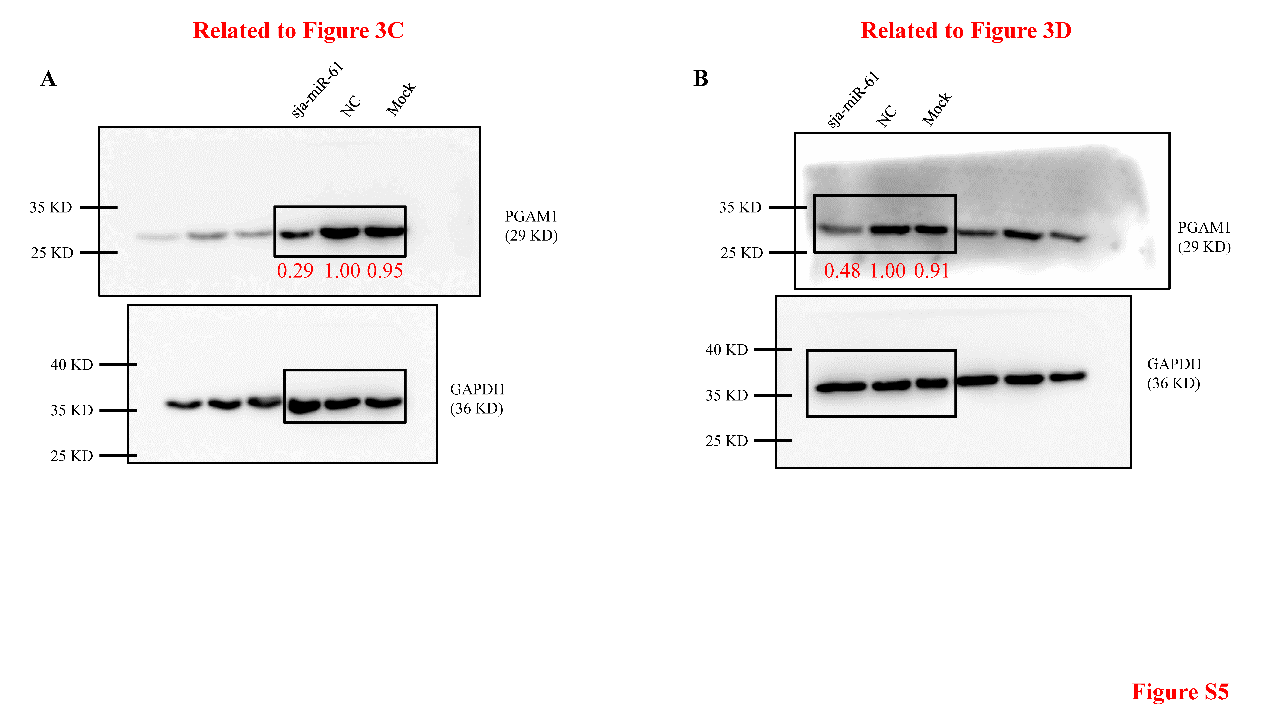
**

**Figure S6. Uncropped western blot images for Figure 3C and 3D.** (A,B) Uncropped western blot images of PGAM1 and GAPDH. A: for Figure 3C; B: for Figure 3D. The bands bordered were used in Figure 3C,D and normalized densitometry ratio denoted in red (divided by GAPDH).

**
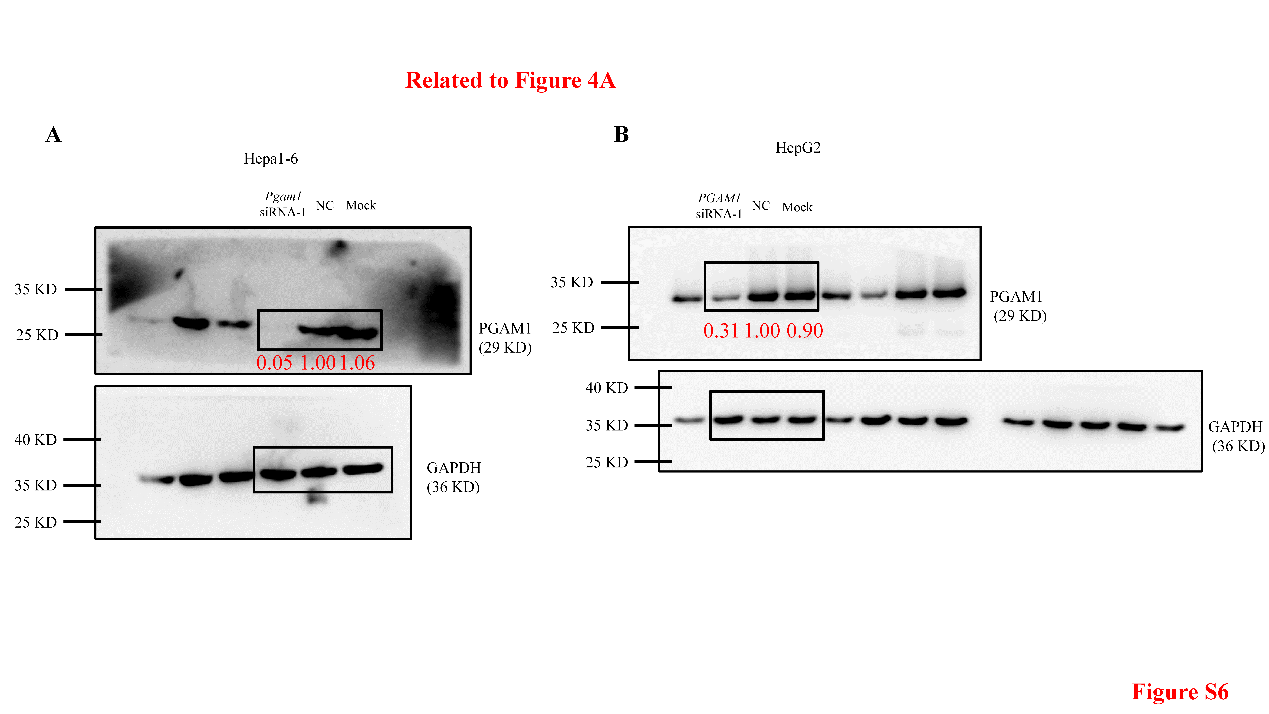
**

**Figure S7. Uncropped western blot images for Figure 4A.** (A,B) Uncropped western blot images of PGAM1 and GAPDH. A: for Hepa1-6 cells; B: for HepG2 cells. Bands bordered were used in Figure 4A and normalized densitometry ratio denoted in red (divided by GAPDH).

**
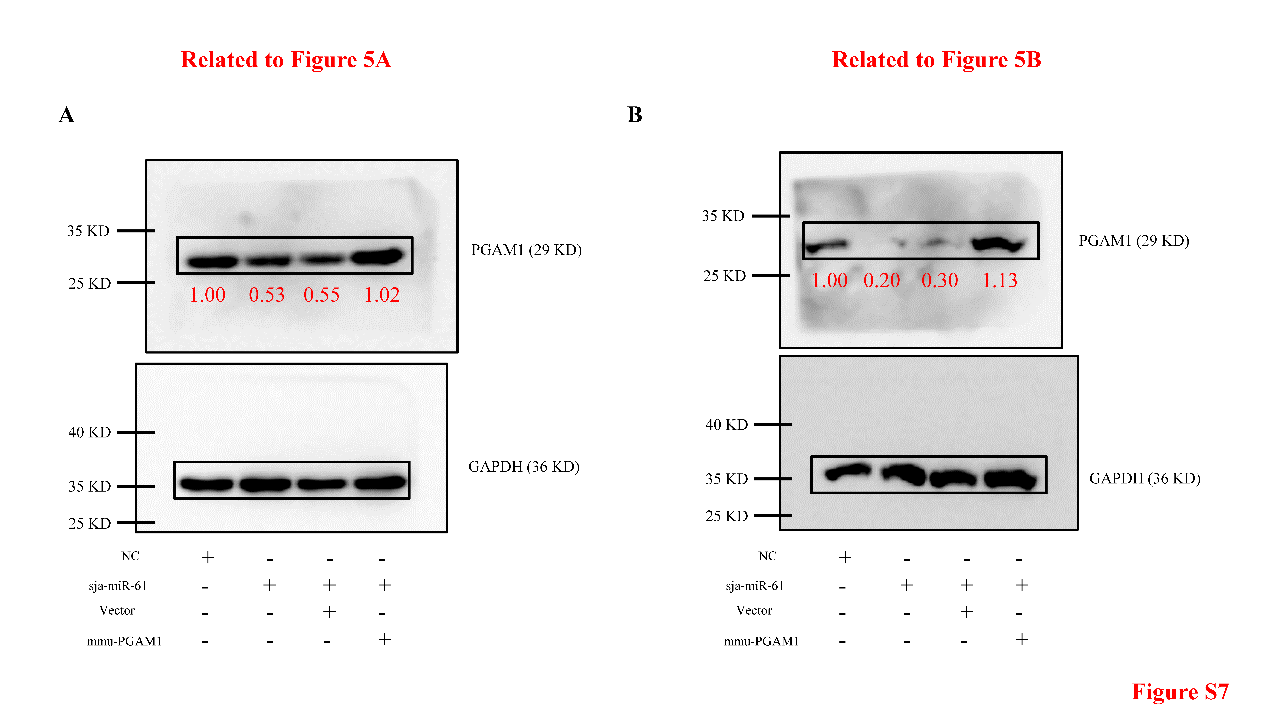
**

**Figure S8. Uncropped western blot images for Figure 5A and 5B.** (A,B) Uncropped western blot images of PGAM1 and GAPDH. A: for Figure 5A; B: for Figure 5B. Bands bordered were used in Figure5A,B and normalized densitometry ratio denoted in red (divided by GAPDH).

**
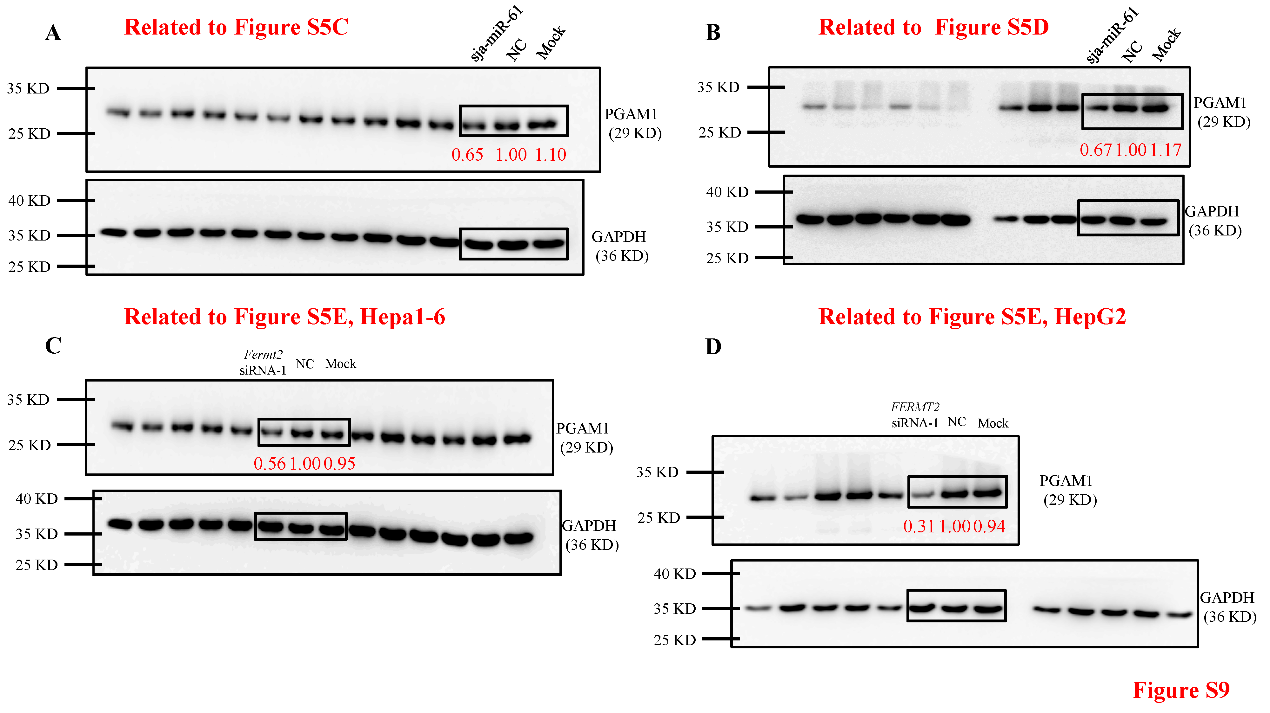
**

**Figure S9. Uncropped western blot images for Figure** **S5C, S5D and S5E.** (A-D) Uncropped western blot images of PGAM1 and GAPDH. A: for Figure S5C; B: for Figure S5D; C and D for Figure S5E. Bands bordered were used in FigureS5C, S5D and S5E, and normalized densitometry ratio denoted in red (divided by GAPDH).

**SUPPLEMENTARY REFERENCES**

1. He X, Xie J, Zhang DM, Su Q, Sai X, Bai RP, et al. Recombinant adeno-associated virus-mediated inhibition of microRNA-21 protects mice against the lethal schistosome infection by repressing both IL-13 and transforming growth factor beta 1 pathways. *Hepatology* (2015) 61(6):2008-17. PubMed PMID: WOS:000354824700027.

2. Zhu SL, Wang S, Lin Y, Jiang PY, Cui XB, Wang XY, et al. Release of extracellular vesicles containing small RNAs from the eggs of Schistosoma japonicum. *Parasite Vector* (2016) 9. PubMed PMID: WOS:000388145300001.
